# Integrated analysis of cell-specific gene expression in peripheral blood using ISG15 as a marker of rejection in kidney transplantation

**DOI:** 10.3389/fimmu.2023.1153940

**Published:** 2023-03-08

**Authors:** Zijian Zhang, Yan Qin, Yicun Wang, Shuai Li, Xiaopeng Hu

**Affiliations:** ^1^ Department of Urology, Beijing Chaoyang Hospital, Capital Medical University, Beijing, China; ^2^ Institute of Urology, Capital Medical University, Beijing, China

**Keywords:** kidney transplantation, rejection, graft survival, monocytes, immune response

## Abstract

**Background:**

Allograft kidney rejection can lead to graft dysfunction and graft loss. Protocol biopsy poses additional risk for recipients with normal renal function. The transcriptome of peripheral blood mononuclear cells (PBMCs) contains tremendous information and has potential application value for non-invasive diagnosis.

**Methods:**

From the Gene Expression Omnibus database, we collected three datasets containing 109 rejected samples and 215 normal controls. After data filter and normalization, we performed deconvolution of bulk RNA sequencing data to predict cell type and cell-type specific gene expression. Subsequently, we calculated cell communication analysis by Tensor-cell2cell and conducted the least absolute shrinkage and selection operator (LASSO) logistic regression to screen the robust differentially expressed genes (DEGs). These gene expression levels were validated in mice kidney transplantation acute rejection model. The function of the novel gene ISG15 in monocytes was further confirmed by gene knockdown and lymphocyte-stimulated assay.

**Results:**

The bulk RNA-seq hardly predicted kidney transplant rejection accurately. Seven types of immune cells and transcriptomic characteristics were predicted from the gene expression data. The monocytes showed significant differences in amount and gene expression of rejection. The cell-to-cell communication indicated the enrichment of antigen presentation and T cell activation ligand-receptor pairs. Then 10 robust genes were found by Lasso regression and a novel gene ISG15 remained differential expression in monocytes between rejection samples and normal control both in public data and animal model. Furthermore, ISG15 also showed a critical role in promoting the proliferation of T cells.

**Conclusion:**

This study identified and validated a novel gene ISG15 associated with rejection in peripheral blood after kidney transplantation, which is a significant non-invasive diagnosis and a potential therapeutic target.

## Introduction

Allogeneic kidney transplantation is a preferred treatment for patients with end-stage renal disease. Rejection severely affects the long-term survival of grafts and recipients and remains a crucial direction in the kidney transplantation field (1–3). However, despite the significant improvement in 1-year allograft survival, approximately 50% of renal allograft recipients experience graft loss within 10-year remaining relatively unchanged in the past decades.

Asymptomatic subclinical rejection occurs in more than 25% of adult kidney transplant recipients within one year after transplantation, while only 6.8% are diagnosed (4). Serum creatinine and blood urea nitrogen are insensitive in identifying subclinical rejection, resulting in delayed response and significantly lower long-term graft survival than non-rejected recipients (3, 5, 6). Therefore, the early diagnosis of rejection is crucial to improve long-term graft outcomes.

Protocol kidney biopsy is a high-accuracy monitoring method for kidney rejection but still limit by time interval and clinical risk (7, 8). Currently, the noninvasive testing strategies to diagnose rejection focus on peripheral blood. Despite lack of accuracy in blood peripheral screening methods, peripheral blood mononuclear cells (PBMCs) RNA sequencing (RNA-seq) contains tremendous information with potential diagnostic value. Several studies described the transcriptome characteristic in kidney allograft recipients with acute rejection (9, 10), chronic allograft nephropathy (11) and antibodies-mediated rejection (12) and proposed potential diagnostic genes for specific types of rejection. However, PBMCs comprise several cell types. Each cellular subtype expresses a unique set of genes, which can reveal critical information about cell identity and state (13); thus, bulk RNA-seq cannot display gene expression in the cells most critical for rejection. Moreover, granulocytes, which account for the largest number of leukocytes, hardly respond to allograft rejection but cause ‘transcriptome noise’ to cover the gene expression differences in immune cells. Single-cell transcriptome sequencing (scRNA-seq) shows the difference in gene expression of each cell type but lacks large sample data for analysis. However, the cost and analysis cycle also limits its application in clinical practice. It is of great potential and importance to search for novel and valid markers in specific cell types of PBMCs for predicting subclinical rejection.

In this study, we collect the gene expression profiles of peripheral blood specimens from 324 renal transplantation patients to seek robust predictive genes associated with rejection. We utilized PBMCs RNA-seq data to perform deconvolution by BayesPrism using a kidney transplant rejection scRNA-seq series as a reference, then enriched our insights into these genes with a cell-cell communication tensor analysis. The novel genes to identify rejection are selected by machine learning. Finally, cell- and animal-based molecular biology experiments confirm a cell-specific novel gene in PBMC as a potential diagnostic and therapeutic target for subclinical rejection. In brief, the present study identified and comprehensively analyzed cell-specific gene expression in peripheral blood, which will contribute to the development of peripheral blood genetic diagnostic techniques after kidney transplantation.

## Materials and methods

### Data collection and preprocessing

We systematically collected 4 datasets containing gene expression profiles of specimens from the Gene Expression Omnibus (GEO) database. Detailed information on all available cohorts is shown in **Table 1**. The gene expression in transplantation PBMCs samples of kidney transplant recipients was performed by bulk RNA sequencing (RNA-seq) or microarray. Microarray expressions were analyzed through “limma” package, while the RNA-seq counts were carried out by the “DESeq2” package running in R 4.3.1. Log2 transformation was performed for all samples.

**Table 1:** Details of 4 datasets included in this study.

Datasets	Techology type	Species	Tissue	Sample	Applications
GSE112927	High-throughput sequencing	Homo sapiens	Blood	235	Discovery of DEGs
GSE120649	High-throughput sequencing	Homo sapiens	Blood	12	Discovery of DEGs
GSE12187	*In situ* oligonucleotide	Homo sapiens	Blood	77	Discovery of DEGs
GSE190329	High-throughput sequencing	Homo sapiens	Blood	4	Reference for deconvolution

### Study design

**Figure 1:**
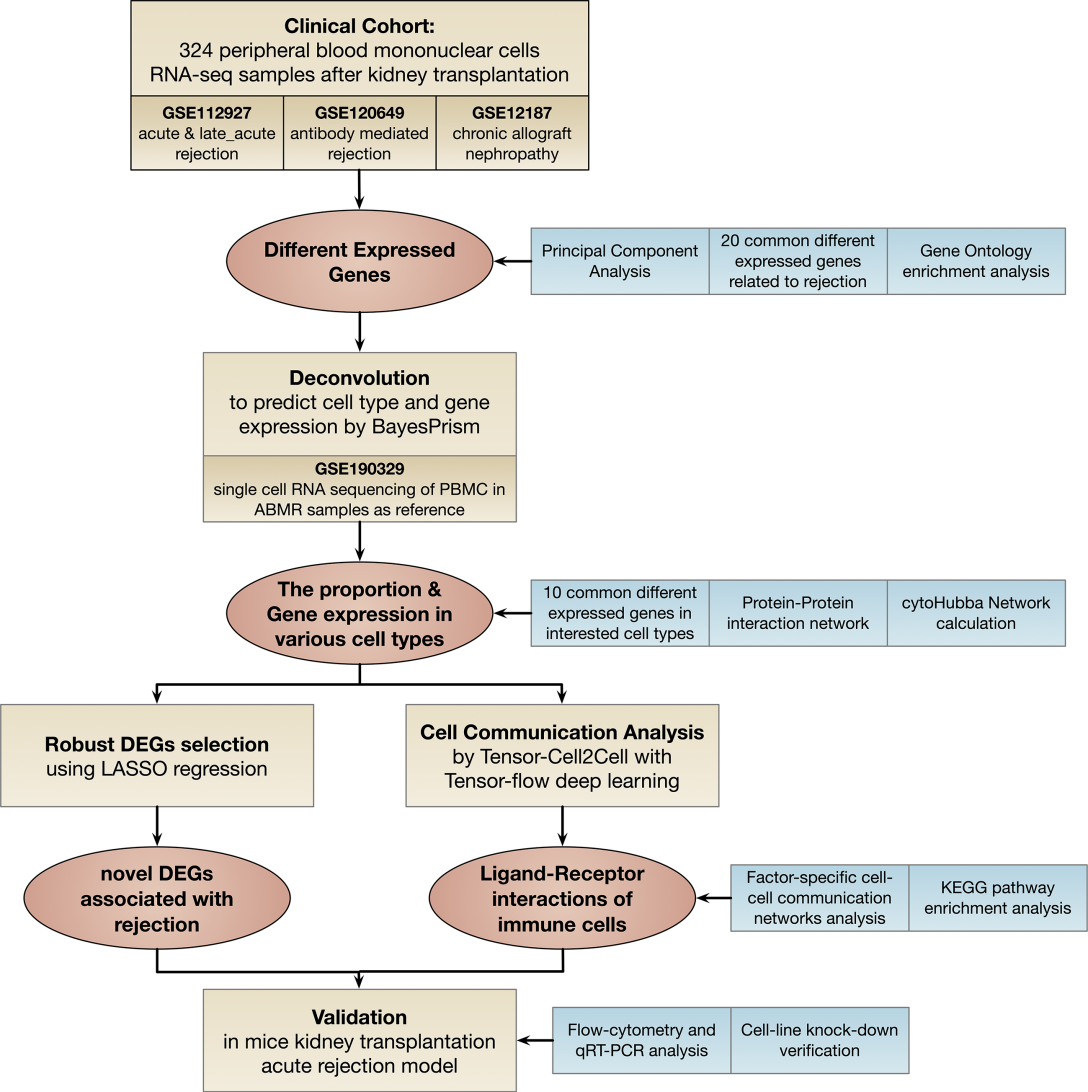
Flowchart of this study. DEGs: different expressed genes; LASSO: least absolute shrinkage and selection operator; KEGG: Kyoto Encyclopedia of Genes and Genomes; qRT-PCR: real-time quantitative reverse transcription polymerase chain reaction.

The flow chart of the study is shown in **Figure 1**. In the clinical cohort, 324 PBMCs RNA-seq samples from patients with acute cellular rejection (ACR), late performed acute cellular rejection (Late_ACR), antibody-mediated graft rejection (ABMR), chronic allograft nephropathy (CAN), and normal control were included to seek common differentially expressed genes (DEGs) related to rejection for further analysis. We performed deconvolution of PBMCs bulk RNA-seq data to predict cell type and cell-type specific gene expression for the downstream analysis. Subsequently, we performed cell communication analysis in PBMCs by Tensor-cell2cell with Tensor-flow deep learning and also conducted the least absolute shrinkage and selection operator (LASSO) logistic regression to screen the robust DEGs further. After systematic profiling, novel identified genes associated with rejection and immune cell intercellular communication were selected for further analysis. These gene expression levels were validated by flow cytometry cell sorting and quantitative real-time polymerase chain reaction (qRT-PCR) in mice kidney transplantation acute rejection model. The function of ISG15 in monocytes was further confirmed by gene knockdown and lymphocyte-stimulated assay.

### Identification and validation of DEGs associated with rejection

Differential expression analyses were performed to identify rejection-related DEGs in peripheral blood. We screened PBMCs RNA-seq samples from ACR, Late ACR ABMR, and CAN. The threshold was set as the adjusted *p*-value <0.01, and the absolute value of log-fold change | log2FC | ≥ 0.4 was statistically significant for the DEGs. DEGs observed in the four rejection types (ACR, Late ACR, ABMR, and CAN) were selected for validation and subsequent analyses.

### Deconvolution of cell type and gene expression

A BayesPrism model estimated the cell type proportion and cell type specific gene expression in the PBMCs RNA-seq datasets (14). The single-cell RNA-seq of PBMC in ABMR samples as reference - were obtained in GSE190329. Default parameters were adopted for the deconvolution analysis.

### Proportion and gene expression in various cell types

According to the preliminary screening of rejection-related DEGs, correlation analysis using protein-protein interaction (PPI) analysis by String and Cytohubba was performed to further identify the DEGs in interested cell types. The PPI network was visualized by Cytoscape software, which can provide researchers with biological network analysis and two-dimensional visualization (15, 16). To further identify the robust expression genes associated with rejection, candidate genes were calculated by LASSO logistic regression using machine learning in Python (Scikit-learn 1.2.0 running in Python 3.9.12). Genes converged by the machine learning method for selection were confirmed as the most relevant genes for rejection after kidney transplantation.

### Cell-cell communication analysis in PBMCs

To infer the intercellular communication networks within PBMCs, Tensor-cell2cell was performed to demonstrate the cell-cell communication patterns (running in Python 3.7). Tensor-cell2cell with tensor-flow deep learning to extract context-driven latent patterns of intercellular communication in an unsupervised manner (14). Besides, the Kyoto Encyclopedia of Genes and Genomes (KEGG) pathway enrichment analysis and factor-specific cell-cell communication networks analysis were also performed to investigate ligand-receptor interactions of immune cells.

### Animal strains and care

C57BL/6 and BALB/c mice (6 to 8 weeks old) were purchased from Vital River Laboratory Animal Technology (Beijing, China). The animals were maintained in a specific pathogen-free facility at the Medical Research Center of Chaoyang Hospital (Beijing, China).

### Kidney transplantation procedure

All donor and recipient mice were housed in a specific pathogen-free (SPF) environment with 12h/12h of light and fed freely. The mouse model for kidney transplantation was established as described (15). Briefly, the donor mice were anesthetized and heparinized. Then the left donor kidney was removed. The same procedure was applied to the recipient, and the blood vessel of the allograft was anastomosed to the recipient’s aorta and inferior vena cava. The ureter was directly implanted into the bladder. Subsequently, rewarmed with warm saline (37°C) and opened the abdominal aorta and inferior vena cava. Following all procedures, the abdominal incision was closed, and postoperative analgesics were administered to aid recovery. No immunosuppression agents were used for an acute rejection model.

### Pathology

The allograft kidney specimens were fixed with 4% paraformaldehyde, dehydrated, embedded in paraffin, and cut into 4-μm sections. Subsequently, hematoxylin-eosin(H&E) staining was performed to assess the kidney allograft rejection. The rejection was confirmed by interstitial inflammation and fibrosis consistent with BANFF 2019 (17).

### Lymphocyte-stimulated assay

To assess the antigen-presenting ability, the dendritic cells were sensitized to allogenous splenocytes for 24 hours, then removed the splenocytes. The target T cell was isolated by FACS, labeled with carboxyfluorescein succinimidyl ester (CFSE, C34570, Invitrogen), and then added to the dendritic cells described previously. 72 hours later, the CFSE signal on T cells was read by flow cytometry.

### Isg15 gene knockdown

The Isg15-targeting and scramble short hairpin RNA (shRNA) sequences were designed using the BLOCK-iT™ RNAi Designer (Thermo Fisher Scientific, Carlsbad, CA) (**Table S1** for sequences). The shRNA sequences were synthesized by Sangon Biotech (Shanghai, China) and cloned into the pLKO.1-mCherry lentiviral vector (128073, Addgene, Watertown, MA, US) and packaged, using Lipofectamine™ 3000 transfection reagent to product recombinant Lenti virus in 293T cell line with coating plasmid psPAX2 and pMD2G. The Hoxb8 transfection immortalized dendritic cell was established following Redecke’s study (18), and then the Isg15 knockdown dendritic cells were established by the Isg15-shRNA lentivirus under Blasticidin S selection.

### Flow cytometry

The animal was harvested 10 days post kidney transplantation surgery, and the PBMCs were isolated using Ficoll-Paque (GE Healthcare, Marlborough, MA). The cells were incubated for CD11b-FITC (561688, BD Pharmingen, San Jose, CA) and F4/80-PE (565410, BD Pharmingen, San Jose, CA) antibodies for 30 minutes at room temperature. The cells were sorted by BD Aria II (BD Bioscience, San Jose, CA)

### Quantitative real-time PCR

Total cellular RNA was extracted using TRIzol reagent (15596026, Invitrogen, Carlsbad, CA) and assessed by qRT-PCR using the SYBR Green mix and Rotor-Gene Q 3 plex system (QIAGEN, Germany). Fold changes in target gene expression were analyzed by Rotor-Gene Q Series Software (QIAGEN, Germany) using the delta/delta CT method. The primers were shown in **Table S2**.

### Western blotting

For western blotting, the cells were harvested and lysis using sample loading buffer (LC1676, Invitrogen). The protein sample was resolved by sodium dodecyl sulfate–polyacrylamide gel electrophoresis and transferred onto 0.45um polyvinylidene fluoride (PVDF) membranes for blotting. After treatment with 5% for blocking, the membrane was incubated with primary antibody anti-Isg15 (15981-1-AP, Proteintech, Rosemont, IL, US) and anti-β-Actin (20536-1-AP, Proteintech), washed and incubated with anti-rabbit horseradish peroxidase (SA00001-2, Proteintech), then incubated in enhanced chemiluminescent SuperSignal West Pico Plus Chemiluminescent Substrate (34580, Invitrogen).

### Statistical analysis

All statistical analyses were performed by R software (version 4.2.1). The D’Agostino and Pearson omnibus normality test was performed to determine if data followed a normal distribution in each comparison. If the data passed the normality test, parametric tests (t-test, one-way ANOVA with Tukey’s correction for multiple comparisons) would be conducted. On the contrary, nonparametric tests were applied (Mann–Whitney-U test, one-way ANOVA using Kruskal–Wallis with Dunn’s correction for multiple comparisons). The *P*<0.05 has been considered statistically significant.

## Results

### Identification of DEGs in kidney transplantation

**Figure 2:**
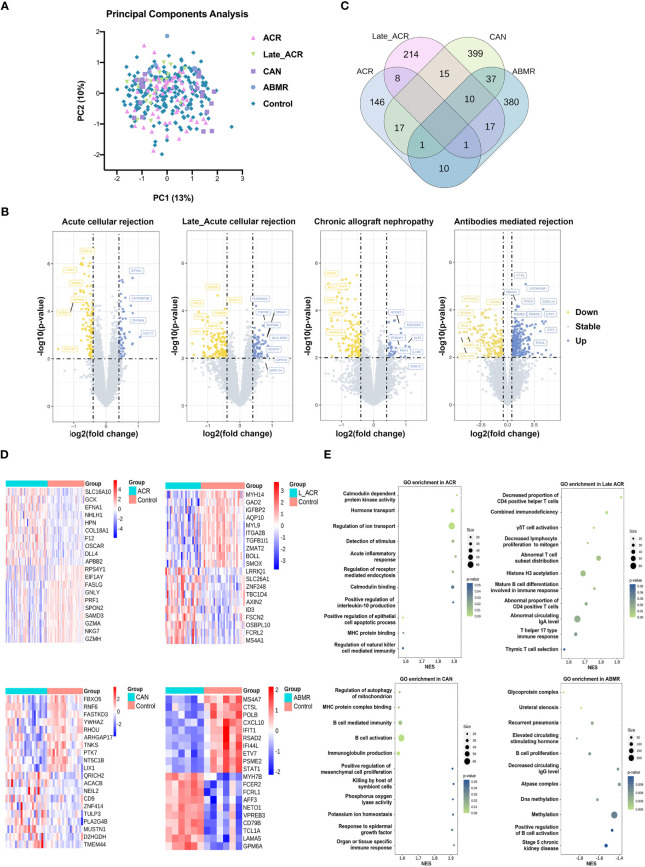
Transcriptome signature of PBMCs for kidney transplantation rejections. **(A)** Principal component analysis indicated the undefined gene expression signature in ACR, late ACR, CAN and ABMR. **(B)** Volcano plots show differently expressed genes between rejected and control samples in each rejection. **(C)** The Venn diagram reveal the common DEGs among groups. **(D)** Clustering heat maps demonstrate expression levels of 20 robust DEGs in different rejection samples. **(E)** The GO pathway enrichment shows the various biological processes in PBMCs. ACR, acute cellular mediated rejection; CAN, chronic allograft nephropathy; ABMR, antibodies-mediated rejection. GO, Gene Ontology.

The transcriptome analysis was performed in PBMC of ACR, late_ACR, CAN, ABMR rejection and normal control, respectively. As illustrated in **Figure 2A**, the principal component analysis indicated a slight difference between rejections and controls, while the volcano plot shows no identical differential expression genes (fold changes > 0.4 and *p*-value < 0.01 as a significant difference, **Figure 2B**). The transcriptome was identified as 89 downregulated and 57 upregulated genes in ACR group, 140 downregulated and 74 upregulated genes in Late-ACR group, 96 downregulated and 303 upregulated genes in CAN group and 246 downregulated and 134 upregulated in ABMR group as DEGs. However, only a small number of genes that are differentially expressed in common across different rejections (**Figure 2C**). The expression of the twenty selected most significant DEGs in these four types of rejection was shown in heatmaps (**Figure 2D**). The GO enrichment revealed representative pathway differences sorted by *p*-values in PBMCs between rejections and controls, including protein kinase activity, protein transport, immune signal, etc. (**Figure 2E**).

### Precise clustering of reference single-cell transcriptome

**Figure 3:**
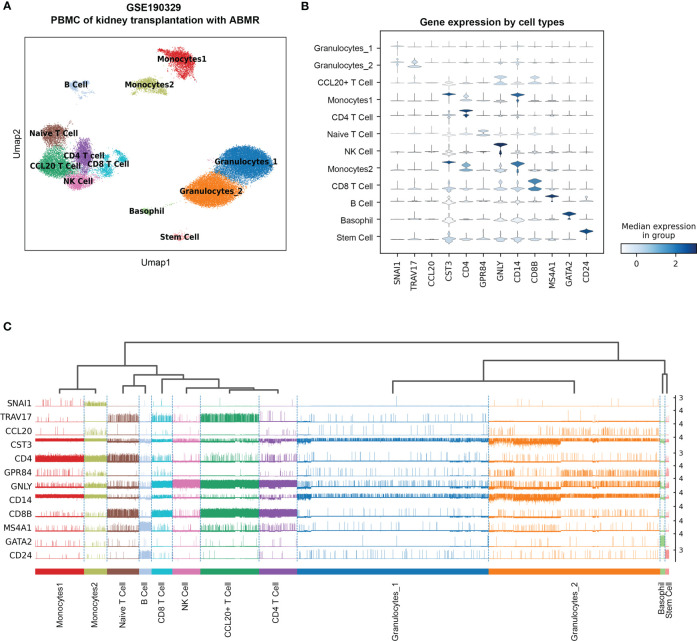
The re-analysis of single-cell RNA sequence data in PBMC with acute antibodies mediated rejection samples as the reference of the deconvolution. **(A)** UMAP dimension reduction analysis of cell type clusters from both Control and cABMR samples. **(B)** The violin plot of marker genes generated by Leiden cluster identify. **(C)** The tracksplot shows the marker genes expression by single-cell resolution grouped by cell types. UMAP: Uniform Manifold Approximation and Projection.

This single-cell transcriptome series was performed from the PBMCs of two cABMR patients and two control patients who did not experience rejection after kidney transplantation. To accurately distinguish cell types in PBMC, ten major cell types and twelve clusters are established based on scRNA transcriptome by a UMAP dimensionality reduction analysis (**Figure 3A**). The cell types include granulocytes, CCL20+ T cells, CD4 T cells, Naïve T cells, NK cells, CD8 T cells, B cells, monocytes, basophils, stem cells, and a small group of other cells (not provided). The violin plot shows the characteristic signature genes for the ten cell populations (**Figure 3B**), while the tracksplot shows the expression of the marker genes at the single-cell resolution (**Figure 3C**). The cell types and gene expression are conversed to a matrix and normalized for subsequent analyses.

### The peripheral immune cell populations and transcriptome characteristics

**Figure 4:**
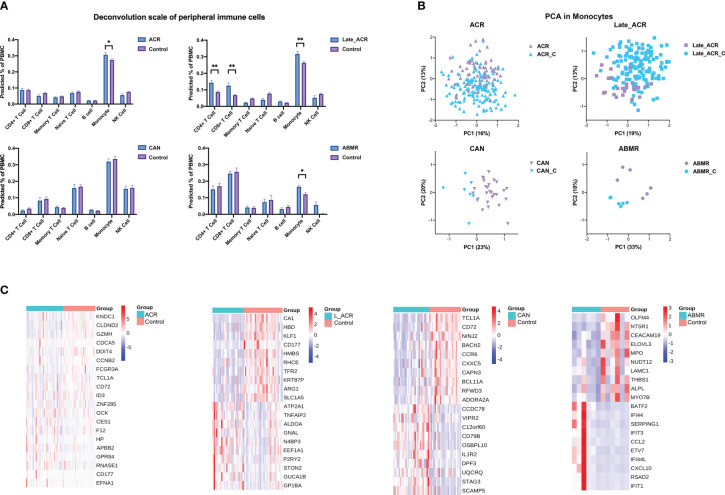
The cell type percentage and gene expression from bulk-RNA seq of rejected and control PBMC deconvolution by BayesPrism. **(A)** The cell type percentage between each rejection group and the control group. **(B)** Principal component analysis indicated the undefined gene expression signature of monocytes in ACR, late ACR, CAN and ABMR group. **(C)** Clustering heat maps demonstrate expression levels of 20 robust DEGs of monocytes in different rejection samples. *p < 0.05 **p < 0.01.

To investigate the changes of immune cells in various rejection samples, a BayesPrism model was trained using the PBMC single-cell RNA sequencing reference as described above to infer proportional cell type fraction and expression level of genes in each cell type from the bulk RNA-seq samples of rejections and controls (**Figure 4**). The reference accommodates multiple gene expression subtypes of the same cell type for a more precise calculation. **Figure 4A** shows seven types of immune cells from PBMC, as expected. The percentage of monocytes was increased in rejection groups except CAN group, while in Late_ACR group, the CD4+ T cells and CD8+ T cells were also elevated (*p* < 0.05). Four PCA plots indicated more robust clusters from deconvolution gene expression levels of monocytes than bulk RNAseq between four types of rejection and control group (**Figure 4B**). Meanwhile, the 20 differential genes in monocytes also showed more significant and more relevant differential expressions analyzed by heatmaps (**Figure 4C**).

### The intercellular communication in immune cell populations

**Figure 5:**
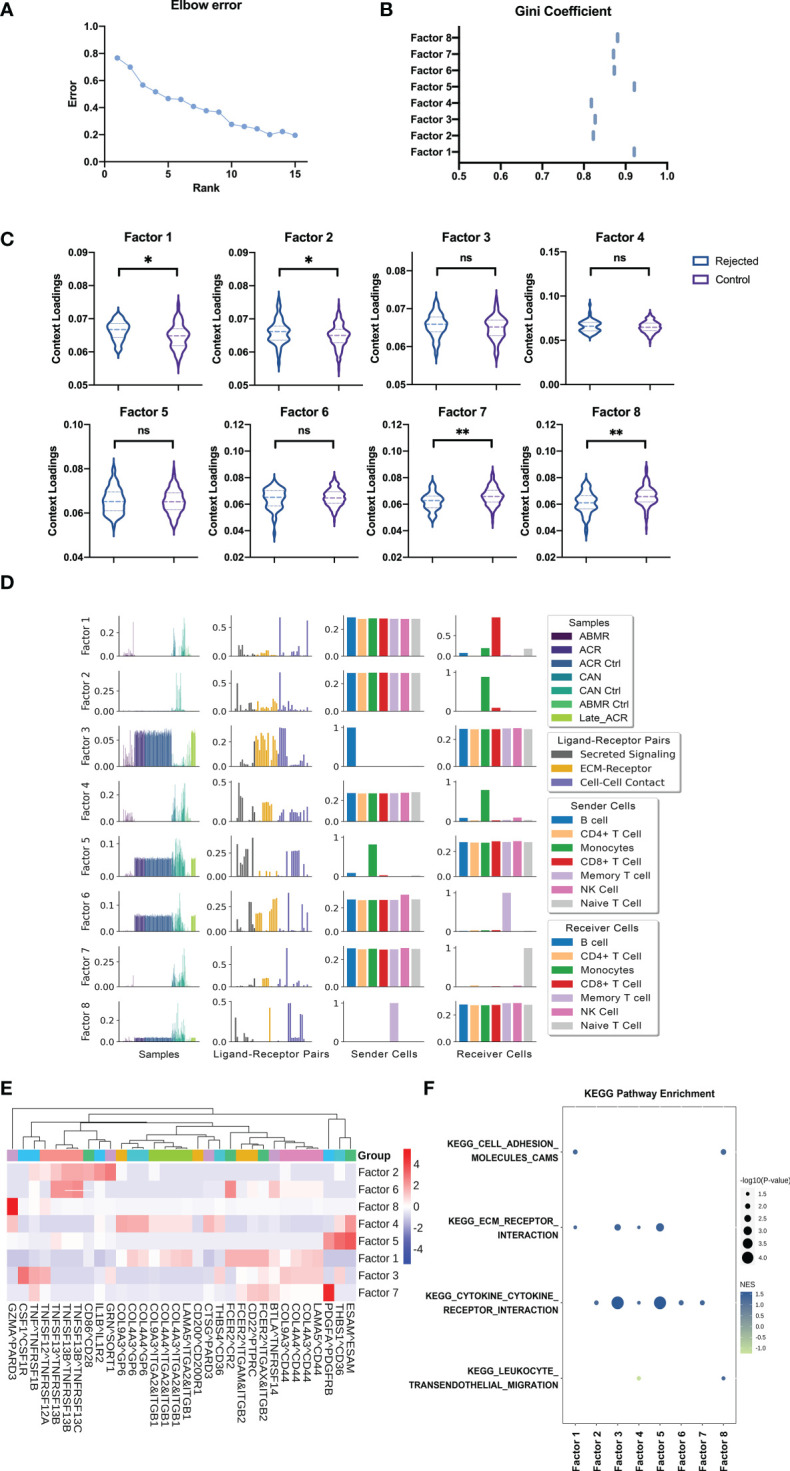
Cell-Cell communication analysis from deconvolution matrix of PBMC RNA sequences. **(A)** The elbow error curve shows the rank number (also represents the number of factors) corresponding to the supervised machine learning efficiency. **(B)** The Gini coefficient quantifies the dispersion of the edge weights in each factor cell communication network (also represents the imbalance of communication). **(C)** Boxplot shows the sample loadings in each of the factors for rejected and control groups of samples. **(D)** Factors by decomposing the tensor from the deconvolution gene expression level of rejected and control patients computing above were selected for the analysis to show the representing dimension in samples, LR pairs, sender cells, and receiver cells. **(E)** Heatmap of the ligand-receptors pairs across factors for integration of the samples, shows the major ligand-receptors pairs contributing to the factors. **(F)** GSEA using the KEGG pathways performed on the LR pairs in each factor. GSEA, Gene Set Enrichment Analysis. "ns" means “not significant”. *p < 0.05 **p < 0.01.

To predict systemic immune cell communication by PBMC, an unsupervised tensor decomposition method named Tensor-cell2cell was used to infer cell communication by the gene expression of the ligand and receptor pairs into a specific sender-receiver cell pair. Then a 4D-communication tensor was generated across different conditions and deconvolved into low-dimensional factors. Eight factors by decomposing the tensor from the deconvolution gene expression level computing above were efficient in evaluating the dimension in ligand-receptor pairs, sender cells, and receiver cells of samples according to the elbow error curve and Gini coefficients (**Figures 5A, B**). After that, four of eight factors can further identify how these functions are associated with different groups, significantly distinguishing the samples with rejection from the normal controls (**Figure 5C**, *p* < 0.05).

The first factor involves the B cell activation with the interactions of other immune cells about the B cell activating factor (BAFF). The receptor-ligand pairs composed of tumor necrosis factor (receptor) superfamily members (TNFSF and TNFRSF) play a key role in activating B cells in transplant rejection, as expected (**Figures 5D, E**). In addition, the receptor-ligand pairs CSF1-CSF1R and IL1B-IL1R2 in factor 2 represent proliferation and activation in the mononuclear macrophage, respectively (**Figures 5D, E**). The naïve T cells are highlighted by Tensor-cell2cell as the primary receiver cells in factor 7, and the molecular mechanisms involving top-ranked signals were provided details as CD200-CD200R1, FCER2-ITGAM-ITGB2, and COL9A3-GP6. The first two suggest the activation signals from monocyte-macrophages to lymphocytes, and the latter implies the adhesion of immature cells (**Figures 5D, E**). In the last, factor 8, the signaling pathway pairs of memory T cells as the sender cells are also related to monocyte-macrophage and co-stimulatory signals. CD86-CD28 is a classic co-stimulatory signaling pathway, while FCER2-ITGAX-ITGB2 may activate tissue-resident memory T cells from transplant organ into the peripheral blood (**Figures 5D, E**).

The cell communication can be further dissected using downstream analyses with standard approaches to run Gene Set Enrichment Analysis (GSEA). Four KEGG pathways obtained the highest enrichment scores regarding cell adhesion, cytokine receptor interaction, and leukocyte migration (**Figure 5F**).

### Precise the signature genes of critical cell types in kidney allograft rejection

**Figure 6:**
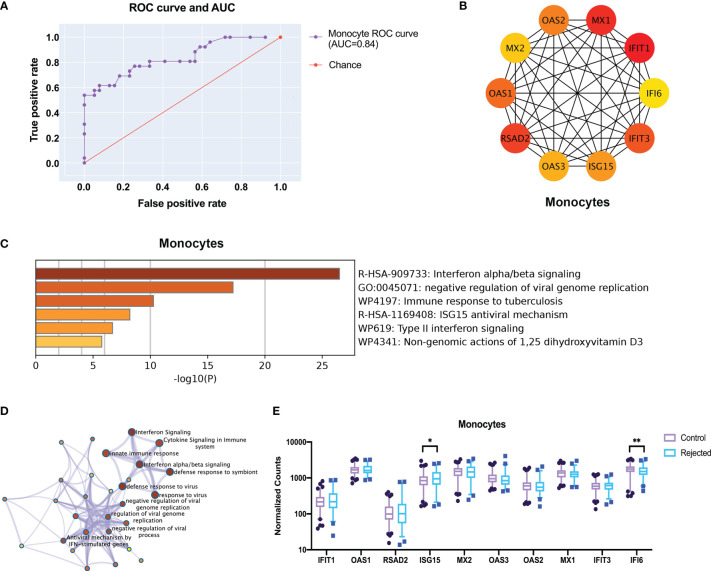
Screen the top 10 robust different expression genes for prediction through transcriptome and protein interactions. **(A)** The ROC curve and AUC show the performance of a classification model at thresholds in all rejection samples of monocytes. **(B)** The protein-protein interactions network shows the 10 hub genes with high connectivity in monocytes after feature selection by LASSO regression. **(C)** The metabolic pathway enriches by GO. **(D)** The network of enriched clusters represents the most significant term of biological process and metabolic pathway. **(E)** The boxplots show the expression of the hub genes in the deconvoluted gene expressions. ROC curve, receiver operating characteristic curve; AUC, area under the ROC curve. *p < 0.05 **p < 0.01.

LASSO regression models with a feature selection function were trained using the deconvoluted gene expression level data to identify the key genes of monocytes. The model can predict the rejection accurately using the gene expression data of peripheral blood cells under certain parameters (**Figures 6A**, **S1**) (alpha=0.01, n_feature=100). These 100 hub genes were further identified as ten strongly connected genes to immune function through the String database and generated into a closer network in accordance with the degree score by CytoHubba (**Figure 6B**). Metascape analysis illustrated clusters of enriched immunology pathways and biological processes, wherein the interferon-alpha/beta signaling cytokines signaling, ISG15 antiviral mechanism and type II interferon signaling in the immune system show the most significant enrichment and most robust connection in monocytes (**Figures 6C, D**). Then these hub genes were verified in gene expression data. Only ISG15 and IFI6 significantly changed in monocytes (**Figure 6E**, *p* < 0.05).

### Verification of novel gene in a mouse kidney transplantation model

**Figure 7:**
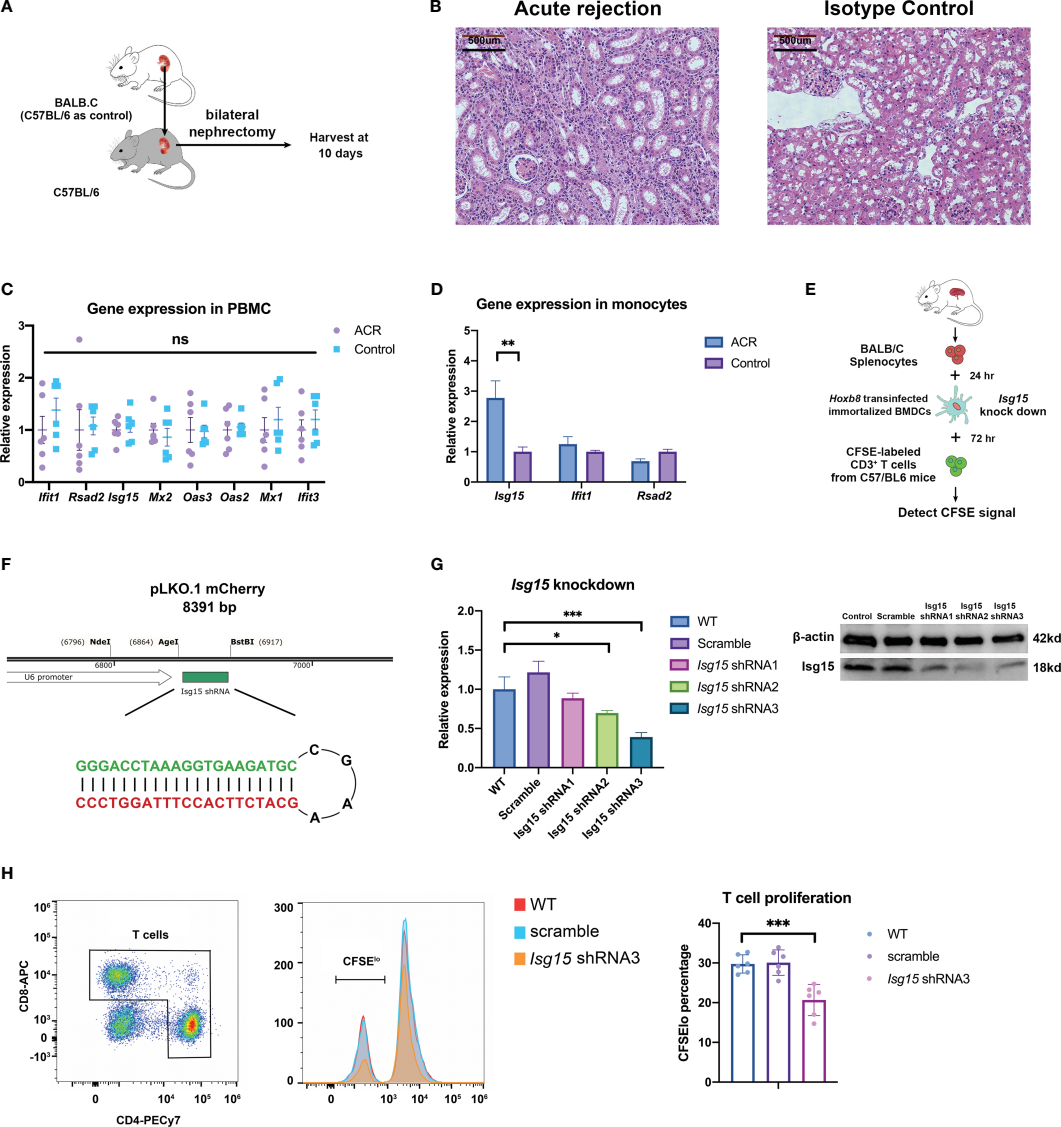
The kidney transplantation experiments verify the correctness and rationality of predicting transplant rejection through gene expression. **(A)** Schematic diagram of acute rejection model in mouse kidney transplantation. **(B)** Pathology of the acute rejection model versus the control group. **(C)** Expression of prediction genes in PBMC between acute rejection and control group. **(D)** Expression of prediction genes in peripheral monocytes isolated by FACS. **(E)** The flowchart of the lymphocyte-stimulated assay. **(F)** Construction strategy for Isg15 gene knockdown by shRNA. **(G)** The knockdown efficiency of Isg15 expression at transcription and translation level. **(H)** The CFSE signal and T cell proliferation in lymphocyte-stimulated assay. FACS, fluorescence-activated cell sorting; CFSE, carboxyfluorescein succinimidyl ester. "ns" means “not significant”. *p < 0.05; **p < 0.01; ***p < 0.001.

Previously, we found that the expressions of ISG15 and IFI6 in monocytes significantly differed in PBMC RNA-seq samples with various allograft kidney rejections (**Figure 6E**). Thus, we established an acute rejection kidney transplantation model in BALB/C and C57BL/6 mice (**Figure 7A**). The histology diagnosis confirmed the acute rejection post-operation for 10 days (**Figure 7B**). The hub gene expression showed no significant difference between ACR and the control group in the whole peripheral blood mononuclear cells (OAS1 and IFI6 had no homologous gene in mice) (**Figure 7C**). However, the Isg15 expression in isolated monocytes of ACR group was increased by nearly three folds (**Figure 7D**). Then we established a functional ex vivo co-culture assay to discover the role of Isg15 in antigen presentation function (**Figure 7E**). The Isg15-shRNAs or scramble shRNA was recombined into the lentivirus interference system to build an Isg15-knockdown dendritic cell model, while Isg15-shRNA3 had the best knockdown efficiency (**Figures 7F, G**, **Table S1**). In this assay, T-cell activation was dependent on antigen presentation by transinfected dendritic cells *in vitro*. Dendritic cells in the control group can stimulate higher levels of T cell proliferation, and knockdown of Isg15 will reduce the antigen presentation of dendritic cells (**Figure 7H**). Therefore, we speculate that Isg15 may be a new potential target for treating kidney transplant rejection.

## Discussion

In recent years, non-invasive diagnostic technique represents a research novelty for predicting rejection after kidney transplantation, which enables earlier intervention, protects kidney function, and indeed prolong graft survival. In our previous studies, we have developed various non-invasive diagnostic techniques based on ultrasonography, magnetic resonance imaging (MRI), and peripheral blood gene signature for predicting early rejection after renal transplantation (19–21). Peripheral blood transcriptome analysis is a promising diagnostic method, but PBMCs contain diverse cells and the bulk RNAseq make it difficult to represent the status of immune cell accurately, which means that the amount and percentage of immune cells rarely changed in various rejections. This study included PBMC transcriptome data from large samples of public databases of patients who experienced rejection after kidney transplantation and normal controls. Principal component analysis (PCA) revealed no significant difference in PBMC transcriptome data between the rejection and control groups. Furthermore, the top DEGs and gene ontology (GO) pathway enrichment also showed evident differences that were irrelevant to the immune response. These indicate that the PBMC transcriptome contains numerous confounding components unrelated to rejection that limit performance in the diagnosis of transplant rejection.

We reanalyzed a small sample of single-cell transcriptome dataset from patients with ABMR after kidney transplantation and normal controls. We identified various types of immune cells from the profiling, including T cells, B cells, monocytes, granulocytes, and NK cells; and we constructed a Bayesian model by using the gene expression signatures of these immune cells as reference. Then deconvolution was performed in a large sample of PBMC transcriptome data to determine the proportion of CD4 T cells, CD8 T cells, and monocytes in various PBMC samples. Based on these analyses, we identified the transcriptome characteristics. Existing computational cell type deconvolution methods are represented as CIBERSORT, EcoTyper, and MuSiC (22–24). In contrast to these deconvolution methods, BayesPrism is an efficient algorithm that utilizes self-defined non-tumor tissues scRNA-seq reference as prior information and infers a joint posterior distribution of cell type proportion and gene expression in each cell type and bulk sample (14). Thus, this work enables us to achieve a deeper comprehension of disease pathogenesis and progression. The results of our analysis demonstrated that the proportion of monocytes increased in renal transplant rejection, the proportion of CD4 T cells and CD8 T cells increased in late ACR, and significant differences were observed in these three cell types at the transcriptome level (**Figure 4**). Moreover, top DEGs were associated with immune response.

We additionally constructed a four-dimension cell-cell communication tensor model to describe the alterations of Ligand-Receptor pairs and cell behavior in peripheral blood immune cells when renal transplant rejection occurred. The results revealed that the differences were mainly manifested in the costimulatory signals of CD86/CD28 dendritic cells (DCs) activated T lymphocytes, GZMA/PARD3 cytotoxic T lymphocyte activation signal pathway, and COL4A3/CD44 cell adhesion and migration signals. These indicated that costimulatory signals of DCs, lymphocytes activation, recirculation and homing were predominant in PBMC in renal transplant rejection. Furthermore, the KEGG pathway enrichment also confirmed the important role of alterations in cytokine receptors, cell adhesion, and migration-related gene expression. Therefore, we selected monocytes for further analysis.

Next, to further screen for promising genes, we constructed a LASSO regression machine learning model, which selected 100 genes with a major contribution to the classifier using feature selection and screened 10 biologically significant hub genes with CytoHubba. Interferon-induced proteins with tetratricopeptide repeats (IFIT) proteins in monocytes mediate cellular immunity and play a role in the immune response and antigen presentation. And, the interferon-stimulated gene 15 (ISG15) is a protein induced by type I interferons and plays an important role in numerous immune functions (25), increased expression of ISG15 in monocytes represents a highly immune stress state and increases the secretion of interferon-r (IFN-r) to further enhance the immune response, which is a potential target for assessing and intervening in immune responses. Besides, GO pathway enrichment for hub genes also suggested an essential role of these genes in innate and adaptive immunity. Eventually, the expression of these genes was validated in transcriptomic data.

Finally, we constructed a kidney transplant acute rejection mouse model to verify the actions of the human-mouse orthologous portions of some of the genes mentioned above in the acute rejection of kidney transplantation. Because IFI6 has no homolog in mice, we only further validated the role of Isg15 in a mouse model. As expected, there was no significant difference in Isg15 or other genes expression in PBMC, whereas Isg15 expression was significantly increased in isolated monocytes in the acute rejection. The ubiquitin-like protein Isg15 is known as a protein induced by type I interferon as a host of antiviral response and immune-evasion strategies in previous studies (26). Isg15 also generates IL-1β-producing CD8α^+^ DCs, which is consistent with the IL1β/IL1R2 ligand-receptor pairs in the previous results. Thus, we selected Isg15 expressed by DCs as the focus for predicting and screening potential therapeutic targets, constructed short hairpin RNA (shRNA) knockdown cell lines in Hoxb8-infected immortalized DCs (18); and used donor spleen cells to activate DCs and stimulate recipient T cells proliferation to mimic the adaptive immune response of ACR. Ultimately, the results found that the knockdown of Isg15 reduced the ability of DCs to activate T cells, which is consistent with a potential target for early diagnosis, prevention, or treatment of ACR.

This study successfully identified and validated a novel gene ISG15 associated with rejection in peripheral blood after kidney transplantation, which is a significant non-invasive diagnosis and a potential therapeutic target. Taken together, we believe that ISG15 lays the foundation for investigating the mechanisms of rejection and is a promising peripheral blood genetic diagnostic technique after kidney transplantation.

## Data availability statement

The original contributions presented in the study are included in the article/**Supplementary Material**. Further inquiries can be directed to the corresponding author.

## Ethics statement

The animal study was reviewed and approved by Beijing Chaoyang Hospital, Capital Medical University.

## Author contributions

ZZ and YQ participated in the research design. ZZ, YW carried out the statistical analysis. ZZ conducted animal experiments. ZZ and YQ drew the figures and drafted the manuscript. YW, SL participated in modifying the manuscript. ZZ, XH provided the funding. All authors contributed to the article and approved the submitted version.
