# Supplementary material for: Integrated analysis of cell-specific gene expression in peripheral blood using ISG15 as a marker of rejection in kidney transplantation
Source: Front Immunol. 2023 Mar 8;14:1153940. doi: 10.3389/fimmu.2023.1153940 (PMC10030514; doi:10.3389/fimmu.2023.1153940)
Supplement: Supplementary file 1 [file DataSheet_1.docx]

Supplementary Material

Integrated analysis of cell-specific gene expression in peripheral blood using ISG15 as a marker of rejection in kidney transplantation

Zijian Zhang^1,2†^, Yan Qin^1,2†^, Yicun Wang^1,2^, Shuai Li^1,2^, Xiaopeng Hu^1,2*^

^1^Department of Urology, Beijing Chaoyang Hospital, Capital Medical University, Beijing, China,

^2^Institute of Urology, Capital Medical University, Beijing, China

*** Correspondence:**Xiaopeng Hu
xiaopeng_hu@sina.com

# Supplementary Figures and Tables

## Supplementary Figures


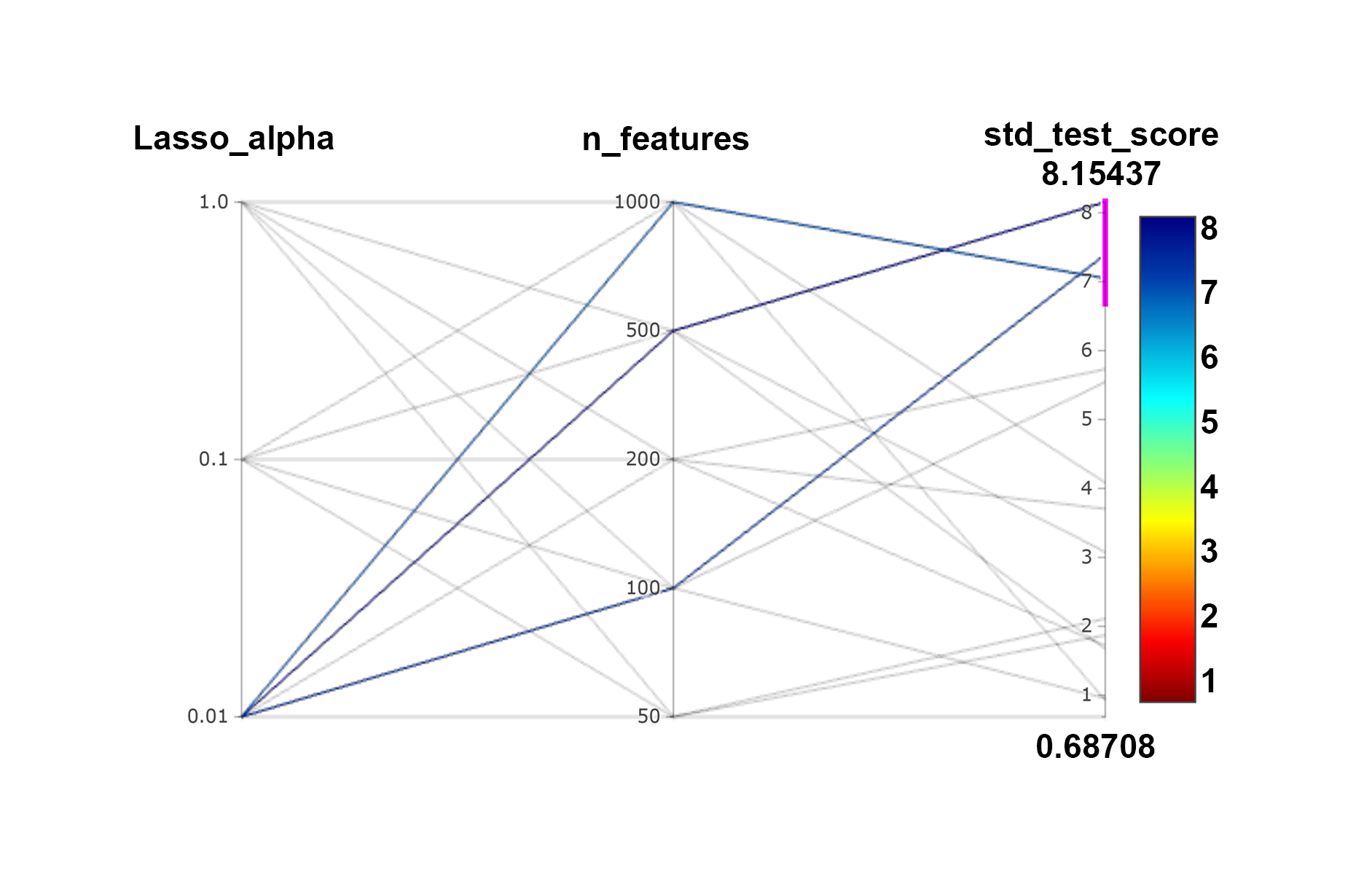


**Supplementary Figure 1.** Hyperparameters and standard test score of LASSO regression.

## Supplementary Tables

| No. | Start | Oligo Sequence |
| --- | --- | --- |
| 1 | 8 | 5'-CCGGTGGGACCTAAAGGTGAAGATGCCGAAGCATCTTCACCTTTAGGTCCCTT-3' |
|  |  | 5'-AGGGACCTAAAGGTGAAGATGCTTCGGCATCTTCACCTTTAGGTCCCA-3' |
| 2 | 200 | 5'-CCGGTGCACAGTGATGCTAGTGGTACCGAAGTACCACTAGCATCACTGTGCTT-3' |
|  |  | 5'- AGCACAGTGATGCTAGTGGTACTTCGGTACCACTAGCATCACTGTGC-3' |
| 3 | 297 | 5'-CCGGTGCAGACTGTAGACACGCTTAACGAATTAAGCGTGTCTACAGTCTGCTT-3' |
|  |  | 5'-AGCAGACTGTAGACACGCTTAATTCGTTAAGCGTGTCTACAGTCTGC-3' |

**Supplementary Table 1.** Isg15-targeting shRNA sequences.

| Gene | Primer sequence |
| --- | --- |
| *Ifit1* | 5'-CAGCAGCACATCTTGCCAAA-3'  5'-GAGGAAGGTGATGCCTGCAA-3' |
| *Rsad2* | 5'-GCCTTGTGTCCCATGACTGA-3'  5'-CACCATGAAGCGTCCCTCTT-3' |
| *Isg15* | 5'-TGGTACAGAACTGCAGCGAG-3'  5'-CAGCCAGAACTGGTCTTCGT-3' |
| *Mx2* | 5'-GTCGCCTATTCACCAGGCTC-3'  5'-TCGTCCACGGTACTGCTTTT-3' |
| *Oas3* | 5'-AGGCTACCGTGTACGCATCT-3'  5'-TTCACACAGCGGCCTTTACC-3' |
| *Oas2* | 5'-AGTGACATGGTGGGAGTGTT-3'  5'-AGCGTCTTCCAGAGCTGAAT-3' |
| *Mx1* | 5'-CGTATCAGAGGGAGACAGCC-3'  5'-CCCGGCCACGATACTGATTT-3' |
| *Ifit3* | 5'-GTGGACTGAGATTTCTGAACTGC-3'  5'-GATTCCCGGTTGACCTCACTC-3' |

**Supplementary Table 2.** Primer sequence for qRT-PCR.
